# Outside in – inside out. Creating focus on the patient – a vaccine company perspective

**DOI:** 10.1080/21645515.2018.1428510

**Published:** 2018-02-15

**Authors:** Denis Sohy, Isabelle Dusart, Philibert Goulet, Diane Visy, Luc Gasthuys, Tatjana Poplazarova, Thomas Breuer, Norman Begg

**Affiliations:** a Senior Publications Lead, GSK Vaccines, Wavre, Belgium; b VoP Coordinator, GSK Vaccines, Wavre, Belgium; c Head of Vaccines Patient Office, GSK Vaccines, Wavre, Belgium; d CRDL Live Viral Vaccines, GSK Vaccines, Wavre, Belgium; e Selling Excellence Director, GSK Vaccines, Wavre, Belgium; f Vice President, Vaccines Research and Development, GSK Vaccines, Wavre, Belgium; g Senior Vice President, Vaccines Research and Development, GSK Vaccines, Wavre, Belgium

**Keywords:** Patient centered, pharmaceutical industry, vaccine, patient engagement, patient focus

## Abstract

Involving patients in the development of medicines and vaccines should result in benefits to patients. The vaccine recipient is usually a healthy person. We describe the rationale and implementation of a vaccine company's initiative to encourage employees to identify with patients of the conditions prevented by the vaccines they help to produce.

The Voice of the Patient (“VoP”), begun in 2014, is an educational programme directed at the 16,000 employees of a global vaccine company. It engages employees through an understanding that they are all “vaccine patients”, and that they can make a difference by considering the impact of decisions made in their day to day work.

The initiative includes presentations about vaccine-preventable diseases, global live webcasts with experts and patients, employee visits to healthcare facilities in developing countries, and the production of patient-focused sections in research publications.

In a 2017 employee survey, 90% of respondents said they know how their daily work impacts patients and they demonstrate focus on patients. We believe this is preliminary evidence that, by supporting employee awareness of the impact of their individual roles, VoP could be a model for a type of initiative that will contribute to industry's continuing evolution towards more patient-centred healthcare.

## Introduction

The concept of patient centricity and engagement in healthcare is generating interest and action among a wide range of stakeholders including academia, governments, funding agencies, regulators, patient groups and the pharmaceutical industry.^1^ Patients are stepping up to have their expertise in healthcare recognized and validated^2^ and their voices heard.^3^ Consumer industries have already shifted their culture to focus on customer needs, now actors in healthcare recognize that the views and experiences of end users can help create and deliver better products and services.^1^ National health regulatory bodies, such as the US Food and Drug Administration and the European Medicines Agency, are incorporating frameworks for engaging patients in their processes. For example, the US FDA's Center for Drug Evaluation and Research holds patient-focused drug development meetings to gain insights from patients into how their particular condition and its available therapies affect their lives. Support for effective patient involvement comes from the continuing development of the Guidance for Reporting Involvement of Patients and the Public (GRIPP2) checklist tools.^4^

Developments in information technology have played a central part in this process. Online availability of information has enabled patients to move from being passive receivers of treatment to playing an active role in making informed healthcare decisions, with or without consultation of Health Care Providers (HCPs). Patient empowerment places a responsibility on healthcare stakeholders to supply them with reliable information. Professional medical journals increasingly recognize the importance of patient readers with the inclusion of lay highlights in their online material. JAMA was a pioneer with its Patient Collections, since 1998;^5^ the BMJ launched its Patient Partnership initiative in 2014;^6^ and the journal Research Involvement & Engagement includes patients in its production, peer review and editorial board.^7^

Patient input is becoming a science in itself.^1^ Patient groups are organized and advocate for research and funding, for example, for rare diseases; and increasingly play a part in drug development and regulation.

Pharmaceutical companies are increasingly recognizing that they share with patients a mutual interest in making available better treatments for medical conditions.^8^ Efforts are being made to achieve a consensus definition of patient centricity, as a step towards consistent patient engagement throughout the product life cycle.^9^ There are opportunities to usefully involve patients at every stage, from developing research questions with real-world applicability, through designing user-friendly consent forms, to participation in post-marketing review.^10^ The science of measuring the benefits of patient and citizen involvement in research and public policy is at an early stage, however,^11^ and although a trend for drug companies to embrace “patient-centricity” without a robust evidence base risks becoming regarded as a fad,^12^ there is a strong ethical imperative^13^ in favour of involving patients in healthcare.

By extension, the potential benefits of partnership with patients also apply to companies that develop and distribute vaccines. In the context of prophylactic vaccination, the “patient” – i.e., the person who is in receipt of prophylactic care by being vaccinated – is most often a healthy person.^10^ Generally speaking, becoming a vaccine recipient is a matter of choice, not a consequence of suffering from a disease. Other people may play a part in – or be impacted by – an individual's decision to vaccinate or not, for example, the prospective vaccinee's parents or another relative or carer. Those impacted range from unborn children to members of the wider community. There is a sense, therefore, in which everybody is a “vaccine patient”. This idea is consistent with a vision of personalized medicine in which the “citizen” participates in the management of their own health, to help prevent disease and promote healthy living.^14^ The use of the term “patient” in the context of vaccination may not, however, be readily understood by employees of a vaccine manufacturer if they intuitively see “patients” as individuals seeking treatment. This presents GSK with a challenge: to create a bridge between vaccine company employees’ day-to-day activities and *Focus on the Patient*, one of the company's leading values.

To meet this challenge, GSK's vaccines division (currently approx. 16,000 employees) developed an initiative that aimed to engage employees through an understanding that they are all themselves “vaccine patients” and that, through their work, they have an opportunity to make a difference to everyone's lives. This paper presents and discusses the conceptual framework and measures agreed by the company, and attempts to demonstrate the impact of steps taken so far.

### The voice of the patient (VoP) initiative at GSK Vaccines

At the start of 2014, the “Voice of the Patient” initiative (VoP) was launched at GSK's vaccine division with its stated aims being to promote a culture of commitment to the patient and to generate an awareness amongst its employees of how their day-to-day activities and decisions have an impact on the vaccine patient. For the purpose of clarity, the initiative used the following definition of patient: “Anyone who may benefit from vaccination now or in the future.”

The original approach was devised by, and continues to be led by, a cross-functional team whose primary activities are in clinical and medical research, manufacturing, quality and commercial divisions. In face to face interviews, 40 members of staff comprising a representative sample of functions, were invited to identify barriers and enablers to patient focus in their work. The most frequently mentioned barrier was the distancing of employees from the patient's experience as a result of lack of direct contact with patients in daily work. Enablers suggested included sharing of patient testimonials, and dedicated representation of patients within the company through a culture of “speaking up for the patient”, and understanding how work functions impact the patient. The interview feedback was used as a basis for a VoP Charter. An identifying message was created for employees:

“I work in vaccines. I know why I am here. I can make a difference to patients. I am a patient.”

**Figure 1.**
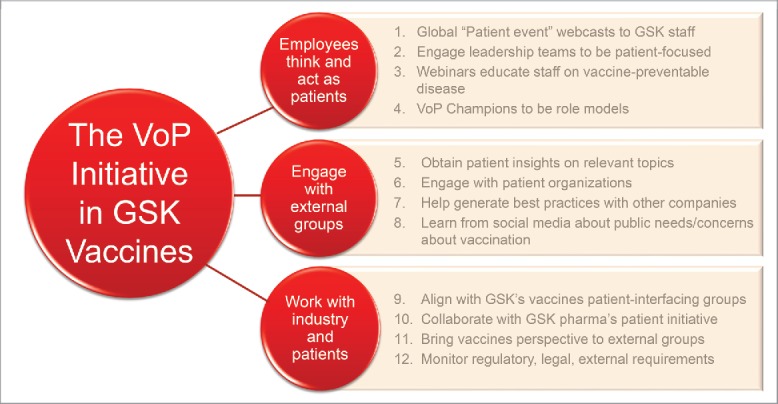
Components of the Voice of the Patient Initiative at GSK Vaccines.

The priorities identified at the outset have been refined over time into the current framework of activities (Fig. 1). Recognising that leadership commitment is needed to achieve real impact,^15^ dedicated staff and appropriate resources have been assigned to VoP within the company, including the establishment of a fully staffed Patient Office in 2016.

**Figure 2.**
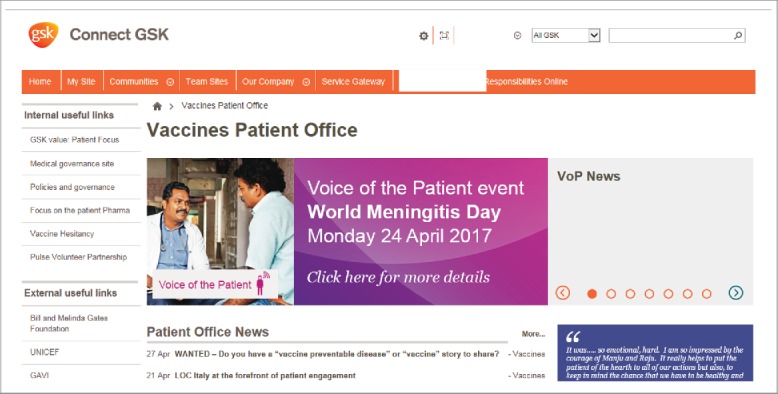
Voice of the Patient (VoP) intranet page for employees of GSK's vaccines division.

By the end of 2014, 122 members of staff from amongst the employee base had volunteered themselves as “VoP Champions”. These volunteers come from different regions/countries and functions and represent various levels of seniority. The Champions promote VoP within their departments through “patient conversations” integrated into existing meetings, supported by videos and slide sets. Global VoP activities include broadcast panel discussions featuring experts and patients from within GSK and externally. These take place during working hours, and attendance is voluntary. A digest sharing published peer-reviewed research into patient engagement, and media reports of patient-centric initiatives undertaken by other entities, is available to all employees via an online workspace (Fig. 2).

Overall quantitative evaluation of the effect of the VoP initiative internally is currently confined to data on levels of employee attendance. In 2016, VoP Champions initiated and held 31 sessions at which employees engaged in discussions on how their work impacts on patients. Global patient events on rabies and meningitis were each attended by over 600 employees (including in person and online), while an intranet report about a session on maternal immunization was accessed by 1905 individuals.

**Figure 3.**
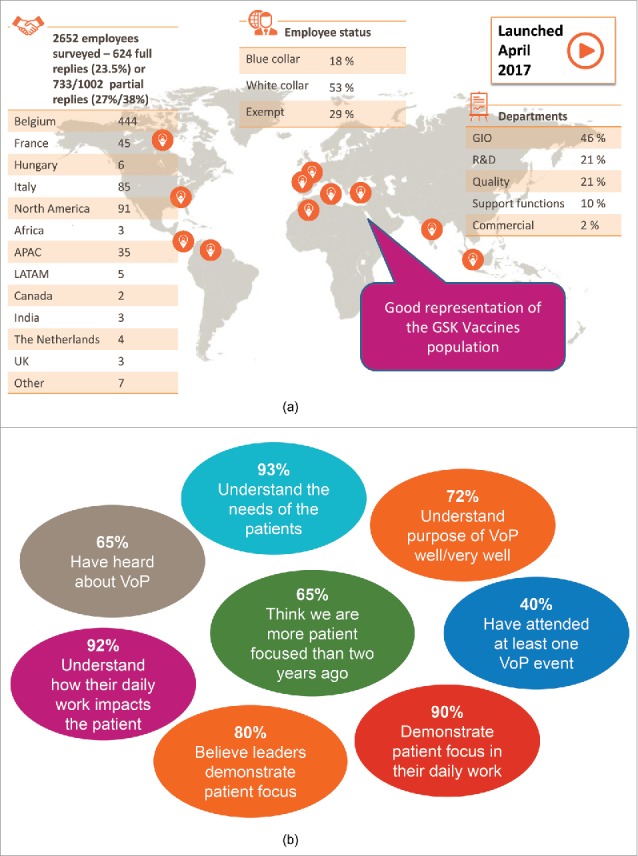
Methodology and results of survey of employees of GSK's vaccines division a) Methodology, b) Survey results.

To assess the impact of the VoP initiative upon the company generally, a survey was sent to 2652 employees of GSK's vaccines division, selected randomly across geographical region, function and seniority. It was available in 6 languages. Response rate was 28%. Analysis of the responses showed that 72% understand that the purpose of the VoP initiative at GSK is to think and act as a patient and for the patient. 65% of respondents feel that GSK focus on patients has increased in the last 2 years, in contrast to 3% who believe that GSK Vaccines do less for the patient than previously. 90% say they know how their job impacts patients, and that they demonstrate patient focus in their daily work. 80% believe that the leaders at GSK Vaccines demonstrate patient focus at work. When asked for suggestions as to how the company could be more patient focused, the most frequent answer was to have more internal communication around the patient (28%). Although survey responses suggest the VoP initiative has delivered its message widely geographically and across all levels of the company, requests were received for the VoP activities to be less focused on the head office and to increase opportunities for involvement of “ground level” employees e.g. manufacturing units (Fig. 3).

Qualitative feedback is systematically collected from employees after patient events and activities, with questions focused on their ability and motivation to integrate the patient perspective when making decisions in their daily working lives. Typical of the responses is a quote received from an employee attending a patient event: “Seeing that the company is investing in encouraging people to focus more on patients is a good source of motivation for me.”

### Some examples of tangible impact of VoP activities among GSK Vaccines employees

#### Webinars – short presentations about vaccine-preventable diseases

Between 2016 and 2017, twenty-three live or online educational sessions have been held on vaccine-preventable diseases in the context of patient focus including rabies, hepatitis A, tick- borne encephalitis, and vaccine hesitancy, reaching more than 2500 employees.

• In a survey of 159 employees who had attended, 89% said that the sessions had been “useful” or “very useful” for their understanding of the disease, and 74% that the presentation motivated them in their day-to-day work.
• 94% of the attendees would advise their colleagues to attend a VoP session.

#### Patient events

Twice-yearly “Patient events” are held comprising a panel discussion between internal or external medical experts and a patient affected by a vaccine-preventable disease. The events are shared with employees globally either on-site or via live webcast. All employees of GSK's vaccines and pharmaceutical divisions have the opportunity to view and take part in the discussion.

• Comment received following a live panel discussion broadcast globally in 2015, which featured a woman whose son had died from congenital rubella syndrome: “I was so impressed by their courage … I realised that whatever our role in the company, we are all acting for the patient at the end of the day. This experience also reminds us to step back, and consider that having the chance to be healthy is key.”

#### Visits to developing countries

The manufacturing department, which processes vaccine batches for supply globally, has sent small groups from its team to Chile and Thailand to meet health care professionals and patients. The visitors comprised members from various functions, including blue- and white-collar workers. They used social media and presentations to share their experiences overseas with their colleagues.

• A manager commented: “My staff could see directly how patients in the areas they visited may be impacted by, for example, supply issues. The visits have had an impact on behaviour, the team feel especially driven to prioritise work connected with timely release of batches of vaccines, to keep the risk of interruption of supply as low as possible.”

#### Patient-focused sections in research publications

GSK's vaccines publications team, which is accountable for the disclosure of results of clinical trials in peer-reviewed journals, has piloted a project involving the systematic production of a “Focus on the Patient” section to accompany manuscript submission, upon author input and approval. This is a brief text summarizing, in an accessible way, the results of clinical relevance to GPs and health care professionals. The journal may choose to publish them in existing journal channels (e.g., “Highlights” or “Take-home messages” on article web pages) or as online supplementary materials. Initial impressions from authors and publications professionals are that the exercise makes them more empathetic towards patient needs.

• As of December 2016, 11 manuscripts have been submitted with highlights sections, and journal editors have responded positively.^16^

#### Employees' own spontaneous initiatives

An employee “brainstorming” session resulted in the proposal that company meetings should begin with the nomination of an employee in the role of “designated patient” who would represent patients’ interests in the matters being discussed.

• A scientific advisor's comment on being designated patient in a Vaccines Commercial Team meeting: “I was amazed to see how many topics could ultimately impact our patients: from deciding the type of syringes, to managing multilingual packaging allocation, to starting up a new bulk facility, to re-evaluating the components of certain vaccines. I would encourage all of us to do this exercise in meetings so that we keep the people we are ultimately serving at the center of our work.”

## Discussion

The VoP initiative encourages employees of GSK's vaccines division to think and act as patients and for patients of vaccine-preventable diseases, in the belief that a focus on the people who use vaccines will result in improved products and services. Responses from employees have been overwhelmingly positive, as measured by the survey and expressed through VoP events' participant feedback.

One of the key messages from the survey on VoP activities concerns the need to increase opportunities for involvement of larger numbers of employees who do not have a direct link with patients in order to bring them closer to the patient dimension (e.g. manufacturing). One can, therefore, propose that a more engaged workforce should ultimately result in benefits for vaccine patients in terms of improved development and delivery of high quality vaccines. Alongside the VoP activities aiming to bring about change within, the newly-established Patient Office will look outwards for opportunities for patient engagement, empowerment and shared participation. Examples include identifying the elements needed for informed decisions on whether to participate in clinical trials, and the development of lay summaries of clinical research results.

A limitation of the initiative results from the difficulty establishing a meaningful baseline for comparison in the absence of pre-initiative survey data. Furthermore, the authors recognize that there is as yet no way to objectively measure the initiative's impact on individuals outside the company environment. Another potential limitation is that patient input into the creation and development of the initiative was limited to that from individuals already within GSK's employ, hence there was no contribution to these aspects from patients external to the company.

The VoP initiative is still evolving and the company believes that it benefits from appropriate leadership commitment and infrastructure to play a useful part in promoting a culture in which patients are respected and involved at all levels, from research planning and design to the delivery of safer and improved products to meet end-user needs in line with the values of GSK – transparency, respect, integrity and patient focus. Further, it would be hoped that sharing the current strengths, limitations and planned developments of the VoP can contribute usefully to the evolution of partnership with patients in healthcare.
